# Radiotherapy for a rare phosphaturic mesenchymal tumor in the middle ear presenting with oncogenic osteomalacia: A case report

**DOI:** 10.1097/MD.0000000000027284

**Published:** 2021-09-24

**Authors:** Taiki Takaoka, Natsuo Tomita, Yoji Shido, Satoshi Baba, Mayu Fukushima, Chikao Sugie, Yuta Shibamoto

**Affiliations:** a Department of Radiation Oncology, Narita Memorial Proton Center, Toyohashi, Japan; b Department of Radiology, Nagoya City University Graduate School of Medical Sciences, Nagoya, Japan; c Department of Orthopedic Surgery, Hamamatsu University School of Medicine, Hamamatsu, Japan; d Department of Diagnostic Pathology, Hamamatsu University School of Medicine, Hamamatsu, Japan; e Department of Radiology, Japanese Red Cross Aichi Medical Center Nagoya Daini Hospital, Nagoya, Japan.

**Keywords:** fibroblast growth factor 23, oncogenic osteomalacia, phosphaturic mesenchymal tumor, radiotherapy, somatostatin receptor 2

## Abstract

**Rationale:**

Phosphaturic mesenchymal tumor (PMT) is a rare neoplasm causing oncogenic osteomalacia. Surgery remains the definitive treatment for PMT, and radiotherapy is seldom employed. However, surgery for PMT involving the head and neck is often difficult due to the local invasion and complicated anatomy. We report the first case of PMT, which was successfully treated with the combination of radiotherapy and supplementation of activated vitamin D.

**Patient concerns:**

A 55-year-old woman suffered from pain in the hip and bilateral femur. Serum phosphate and calcium decreased to abnormal levels. Serum alkaline phosphatase and fibroblast growth factor 23 increased to abnormal levels. The hearing loss of the right ear had continued and a middle ear tumor was revealed.

**Diagnoses:**

Subsequent biopsy provided the diagnosis of PMT that caused oncogenic osteomalacia. These clinical and pathological characteristics were consistent with and provided the final diagnosis of benign PMT.

**Interventions:**

Surgery of the PMT was difficult and the patient underwent radiotherapy. The prescribed dose was 36 Gy in 10 fractions. Simultaneously, the patient started supplementation of 1,25-dihydroxyvitamin D3 (1–2 μg/day) and continued for 2 years.

**Outcomes:**

Near-complete resolution of the symptoms was achieved and abnormal laboratory values recovered. At 5 years of follow-up, the irradiated tumor showed no regrowth. Severe hearing loss of the right ear was not observed.

**Lessons:**

Radiotherapy was effective for the PMT and could be an important treatment option for inoperable cases.

## 1 Introduction

Phosphaturic mesenchymal tumor (PMT) is a rare neoplasm causing oncogenic osteomalacia characterized by increased renal phosphate excretion and hypophosphatemia.^[1]^ PMTs arising in the head and neck are extremely rare, mostly in the extremities and appendicular skeleton.^[2,3]^ Although surgery remains the definitive treatment, radiotherapy is an alternative treatment for inoperable patients. So far, there have been no reports of successful treatment of PMT with radiotherapy. We herein report a patient presenting with oncogenic osteomalacia due to a PMT in the middle ear, who was successfully treated with the combination of radiotherapy and supplementation of activated vitamin D.

## 2 Case report

**Figure 1:**
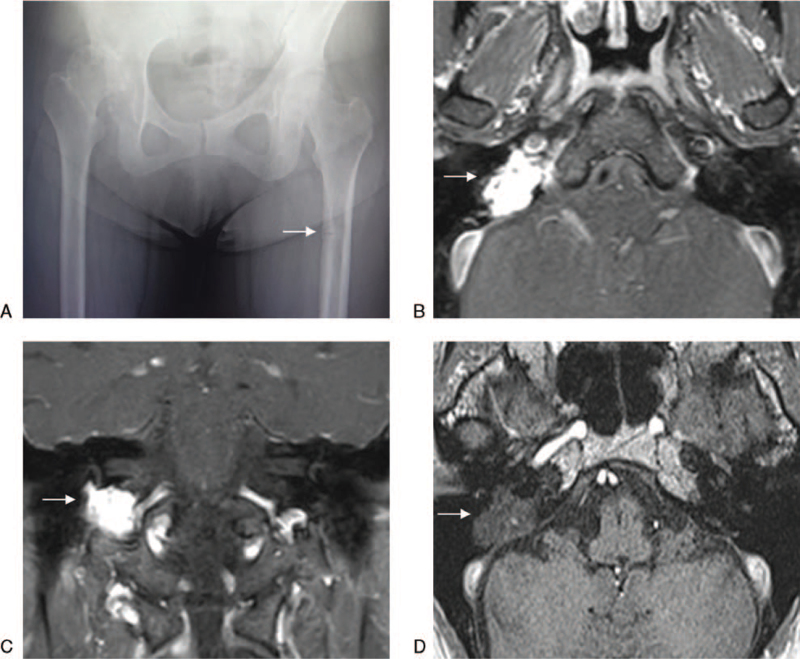
A: Plain radiography of the hip and femur. The arrow represented the mild radiolucent shadow. B: Transverse magnetic resonance imaging (MRI) before radiotherapy, gadolinium-enhanced fat saturation T1-weighted. The arrow represented the tumor and the size was 1.5 × 1.5 × 1.0 cm. C: Coronal MRI before radiotherapy, gadolinium-enhanced fat saturation T1-weighted. The arrow represented the tumor and local invasion. D: Transverse MRI at 5 years after radiotherapy, non-enhanced T1-weighted. The arrow represented the irradiated tumor.

**Figure 2:**
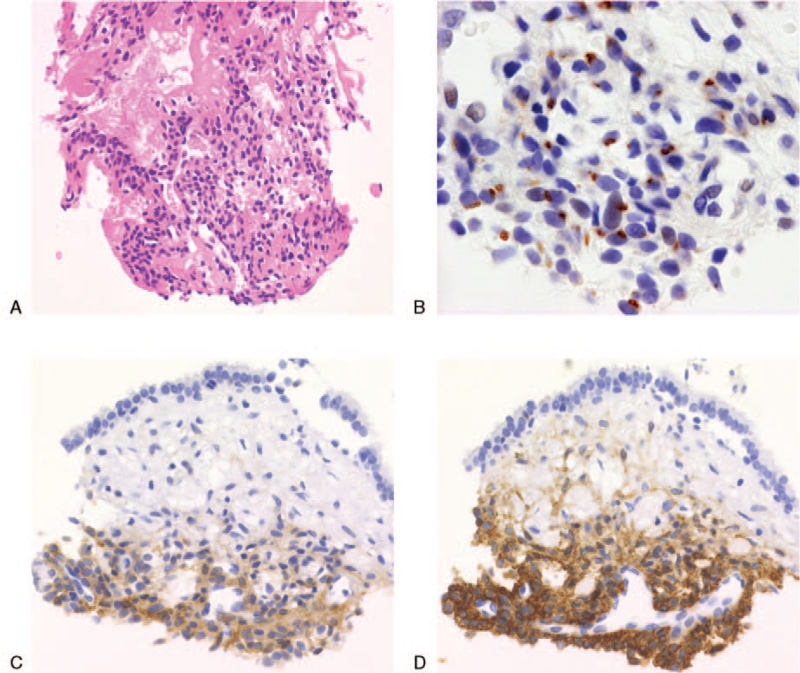
A: Short spindle-shaped cells proliferating around the branched microvessels presented a hemangiopericytoma-like growth pattern. Hematoxylin and eosin staining (×40). B: Tumor cells showed positive expressions of fibroblast growth factor 23, a dot-like pattern (×100). C: Tumor cells showed positive expressions of somatostatin receptor 2 (×40). D: Tumor cells showed positive expressions of cluster of differentiation 56 (×40).

A 55-year-old woman visited an orthopedic surgery department with a complaint of pain in the hip and bilateral femurs. There was no history of trauma, and abnormal findings on plain radiography were not identified except for a mild radiolucent shadow in the left femur (Fig. 1A). In additional magnetic resonance imaging (MRI), a linear hypointensity consistent with the mild radiolucent shadow in the left femur was detected on T1-weighed images and provided the diagnosis of occult fracture. The blood test revealed decreases in serum phosphate and calcium levels to 2.2 mg/dL and 8.4 mg/dL, respectively. The additional blood test revealed significant increases in serum alkaline phosphatase and fibroblast growth factor 23 (FGF23) levels to 535 U/L and 1140 pg/mL (normal range < 30 pg/mL), respectively. The hearing loss of the right ear had continued for many months before the occurrence of pain in the hip and bilateral femurs, so the patient had an otolaryngological consultation at the same time. Otoscopic examination and MRI of the cerebellopontine angle revealed a mass in the right middle ear, measuring 1.5 × 1.5 × 1.0 cm in the largest dimensions (Fig. 1B, C). Subsequently, a biopsy of the mass was performed for histological diagnosis. Necrosis and mitotic figures indicating malignancy were absent. Short spindle-shaped cells proliferating around the branched microvessels presented a hemangiopericytoma-like growth pattern (Fig. 2A). Tumor cells stained positive for FGF23 (Fig. 2B), somatostatin receptor 2 (Fig. 2C), and cluster of differentiation 56 (Fig. 2D) but negative for h-caldesmon on immunohistochemistry. The pathological diagnosis was PMT. These clinical and pathological characteristics were consistent with and provided the final diagnosis of benign PMT.

**Figure 3:**
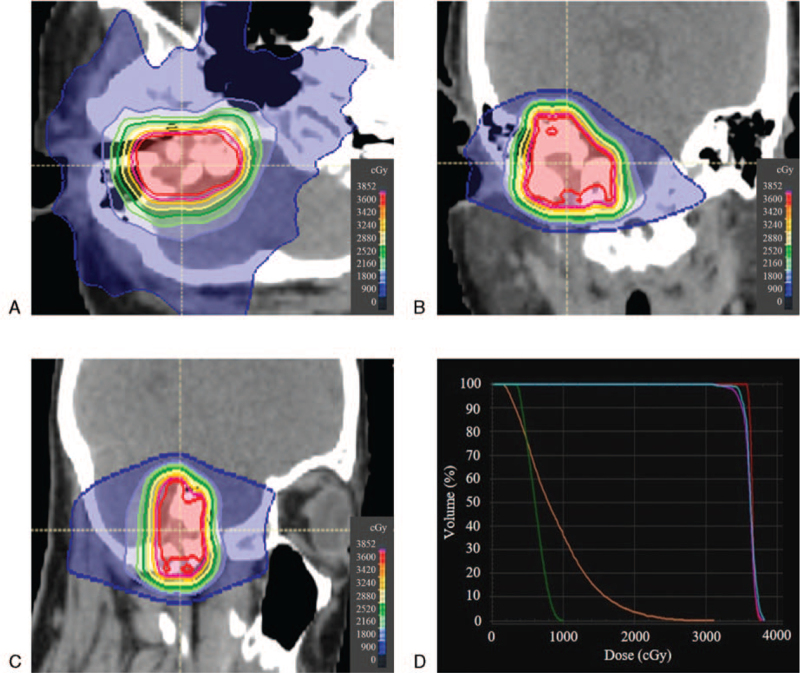
Radiotherapy planning using helical tomotherapy. A: Isodose distribution in the transverse plane. B: Isodose distribution in the coronal plane. C: Isodose distribution in the sagittal plane. D: Dose volume histogram. Red, pink, blue, green, and orange lines represented gross tumor volume, planning target volume, right inner ear, left inner ear, and brain stem, respectively.

The patient underwent radiotherapy because surgery was difficult due to the local invasion. The gross tumor volume (3.8 cm^3^) was the visible lesion on MRI. Taking the direction of tumor invasion into consideration, the clinical target volume (10.8 cm^3^) was adjusted based on anatomical structures. The planning target volume (20.9 cm^3^) was defined with a 3-mm margin around the clinical target volume. Subsequent planning and treatments were carried out with the Tomotherapy version 5.0.1 treatment planning station and TomoTherapy HDA system (Accuray, Inc, Sunnyvale, CA). The TomoHelical mode was employed. The prescribed dose was 36 Gy in 10 fractions over 2 weeks once a day to cover 50% of the planning target volume. The field width, pitch, normal modulation factor, and irradiation time was 1.0 cm, 0.43, 2.0, and 5.6 minutes, respectively. Our method of helical tomotherapy was previously described in detail.^[4]^ The constraints for normal organs were equal to the tolerance dose of normal tissue in radiotherapy.^[5]^Figure 3 showed the dose distributions and dose volume histogram of radiotherapy. Radiotherapy was performed with no acute toxicity. Simultaneously, the patient started supplementation of 1,25-dihydroxyvitamin D3 (1–2 μg/day) and continued for 2 years.

**Figure 4:**
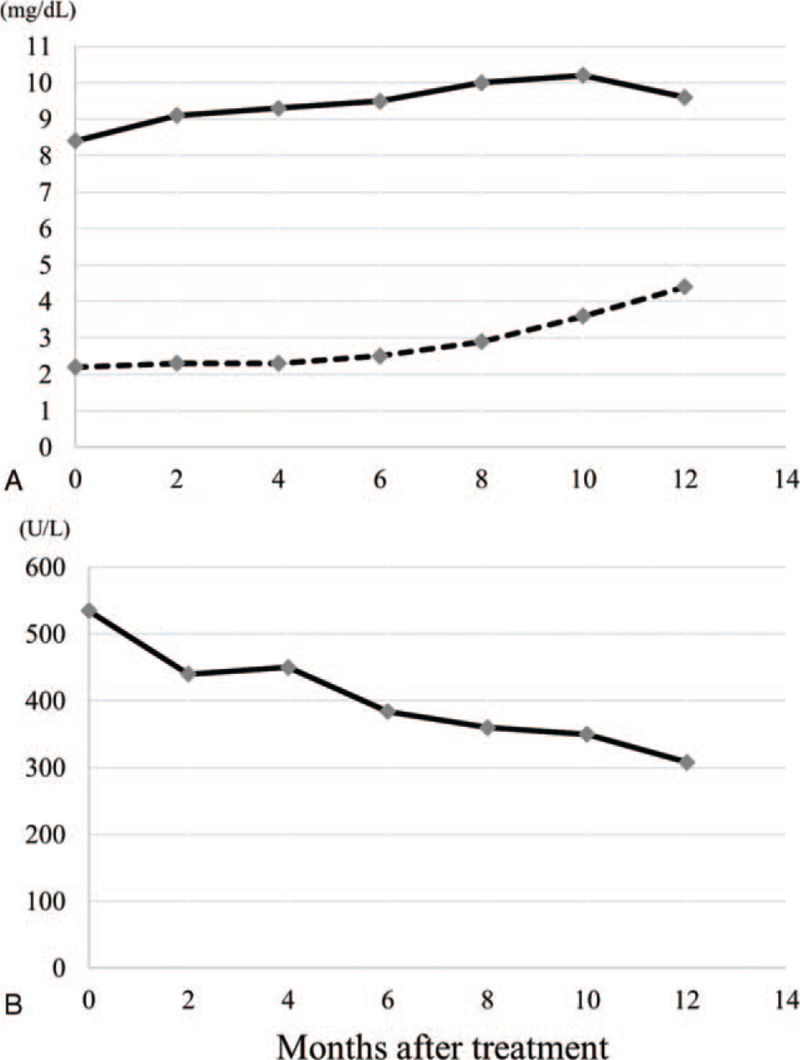
Changes in serum calcium, phosphate, and alkaline phosphatase after treatment. A: The solid line represented the changes in serum calcium (normal range, 8.7–10.3 mg/dL). The broken line represented the changes in serum phosphate (normal range, 2.5–4.7 mg/dL). B: The solid line represented the changes in serum alkaline phosphatase (normal range, 115–350 U/L).

After radiotherapy, the patient was followed every 2 months during the first year, and at intervals of 6 or 12 months thereafter. Regular follow-up included pure-tone audiometry, blood tests, and MRI. At 2 months of follow-up, serum calcium recovered dramatically owing to the supplementation of 1,25-dihydroxyvitamin D3. At 8 months, normalization of serum phosphate and alkaline phosphatase levels was achieved and maintained thereafter. Figure 4 showed changes in serum calcium, phosphate, and alkaline phosphatase after treatment. At 2 years, the patient achieved near-complete resolution of bone pain. At 5 years, repeat MRI showed no regrowth of the irradiated tumor (Fig. 1D). Concerning the hearing acuity of the right ear, the Gardner-Robertson scale was used to evaluate the hearing level. Pre-treatment hearing level was scale II (38 dB). Posttreatment hearing level was scale II (40 dB) at 1 year of follow-up, scale II (46 dB) at 2 years, scale II (48 dB) at 3 years, scale III (56 dB) at 4 years, and scale III (52 dB) at 5 years, respectively. No other chronic toxicities were observed.

## 3 Discussion

Oncogenic osteomalacia is known as a rare and curable cause of osteomalacia characterized by increased renal phosphate excretion and hypophosphatemia.^[1]^ PMT is a rare neoplasm arising in bone and soft tissue that inappropriately produces FGF23, which has phosphaturic activity inhibiting renal tubular reabsorption of phosphate and renal conversion of 25-hydroxyvitamin D3 to 1,25-dihydroxyvitamin D3 (active form of vitamin D). This results in hypophosphatemia and ultimately bone loss.^[6]^ PMT is considered the most common cause of oncogenic osteomalacia.^[1]^ PMTs arising in the head and neck are extremely rare, mostly in the extremities and appendicular skeleton.^[2,3]^ PMT is usually benign but the local invasiveness is a characteristic feature.^[3]^

The definitive treatment for oncogenic osteomalacia is surgical resection of the causative PMT,^[7,8]^ and radiotherapy is seldom employed. However, surgery for PMT involving the head and neck is often difficult due to the local invasion and complicated anatomy,^[9]^ as the present case exhibited. Radiotherapy should be considered as an alternative treatment for inoperable cases. Cases of PMTs treated with radiotherapy were summarized in Table 1.^[10–12]^ It was reported postoperative radiotherapy for benign PMT was effective to improve oncogenic osteomalacia. Although the detailed mechanism of the effectiveness of radiotherapy for benign PMT was still unclear, the obstruction and fibrosis of the tumor vessels could occur thus inhibiting the growth, similar to those observed in other hormone- or cytokine-producing tumors.^[13,14]^ Considering the mechanism, radiotherapy could take some time to resolve the symptoms compared with surgery. Although PMTs and acoustic tumors were different types of tumors, the tumor location and surrounding normal tissues of the present case were similar to acoustic tumors. Considering the PMT was benign tumor, the employed dose was decided based on the excellent local control rates of acoustic tumors.^[15,16]^ We considered the dose was probably enough to control benign PMT. It was considered that the employed dose was appropriate to prevent severe toxicities of normal organs around the target. Various studies on fractionated stereotactic radiotherapy for acoustic tumors have been reported in literature.^[17,18]^ Especially in hearing outcomes, it was reported that the mean cochlear dose <40 Gy was a significant factor associated with hearing preservation.^[17]^ Indeed, severe hearing loss was not observed in the present case. Malignant transformation from benign PMT was reported in the long-time follow-up.^[19]^ Therefore, we will carefully continue the follow-up of the patient.

**Table 1:** Cases of phosphaturic mesenchymal tumor treated with radiotherapy.

Case	Author	Age, gender	Location	Pathology	Surgery	Radiotherapy	OO
1	Shah et al^[10]^	60, male	Ethmoid sinus	Benign PMTMCT	Endoscopic resection	Postoperative, 54 Gy/30fxs	Cured
2	Lee et al^[11]^	52, male	Ethmoid sinus	Benign PMTMCT	Endoscopic resection	Postoperative, unknown dose	Cured
3	Uramoto et al^[12]^	48, male	Tongue	Malignant PMTMCT	Marginal resection	Salvage, 66 Gy/33fxs	Cured

fxs = fractions, OO = oncogenic osteomalacia, PMTMCT = phosphaturic mesenchymal tumor, mixed connective tissue variant.

## 4 Conclusion

This was the first case report showing successful treatment of benign PMT with the combination of radiotherapy and supplementation of activated vitamin D. This report would serve to increase the awareness of this uncommon disease and could be a reference for employing radiotherapy in future inoperable cases.

## Acknowledgment

We would like to thank all staffs at Narita Memorial Hospital and our hospitals.

## Author contributions

TT contributed to collect data and draft the manuscript. NT contributed to collect data and revise the manuscript. YS contributed to the treatment of oncogenic osteomalacia. SB contributed to the pathological diagnosis of phosphaturic mesenchymal tumor. MF contributed to the staining of histopathology specimens. CS contributed to generate the radiotherapy planning. YS contributed to translate and revise the manuscript. All authors read and approved the final manuscript.

**Data curation:** Taiki Takaoka, Natsuo Tomita, Yoji Shido, Satoshi Baba, Mayu Fukushima, Chikao Sugie.

**Investigation:** Yoji Shido, Satoshi Baba, Mayu Fukushima, Chikao Sugie.

**Writing – original draft:** Taiki Takaoka.

**Writing – review & editing:** Natsuo Tomita, Yuta Shibamoto.
